# Blood acid-base status in impala (*Aepyceros melampus*) immobilised and maintained under total intravenous anaesthesia using two different drug protocols

**DOI:** 10.1186/s12917-017-1163-8

**Published:** 2017-08-16

**Authors:** Gareth E. Zeiler, Leith C. R. Meyer

**Affiliations:** Department of Paraclinical Science, Faculty of Veterinary Science, University of Pretoria, Private Bag X04; Onderstepoort, Pretoria, Gauteng 0110 South Africa

**Keywords:** Blood pH, Impala, *Aepyceros melampus*, Immobilisation, General anaesthesia, Henderson-Hasselbalch, Stewart approach

## Abstract

**Background:**

In mammals, homeostasis and survival are dependent on effective trans-membrane movement of ions and enzyme function, which are labile to extreme acid-base changes, but operate efficiently within a narrow regulated pH range. Research in patients demonstrating a pH shifts outside the narrow regulated range decreased the cardiac output and systemic vascular resistance and altered the oxygen binding to haemoglobin. These cardiopulmonary observations may be applicable to the risks associated with anaesthesia and performance of wildlife ungulates on game farms. The aim of this study was to compare blood pH changes over time in impala immobilised and anaesthetised with two different drug protocols (P-TMP - immobilisation: thiafentanil-medetomidine; maintenance: propofol-ketamine-medetomidine; P-EME – immobilisation: etorphine-medetomidine; maintenance: etorphine-ketamine-medetomidine). Additionally, we discuss the resultant blood pH using both the Henderson-Hasselbalch and the Stewart approaches. Two data collection time points were defined, Time1 before maintenance of general anaesthesia and Time 2 at end of maintenance of general anaesthesia. We hypothesise that blood pH would not be different between drug protocols and would not change over time.

**Results:**

Significant differences were detected over time but not between the two drug protocols. Overall, the blood pH decreased over time from 7.37 ± 0.04 to 7.31 ± 0.05 (*p* = 0.001). Overall, over time arterial partial pressure of carbon dioxide changed from 51.3 ± 7.5 mmHg to 72.6 ± 12.4 mmHg (*p* < 0.001); strong ion difference from 44.6 ± 2.4 mEq/L to 46.9 ± 3.1 mEq/L (*p* < 0.001); anion gap from 15.0 ± 3.1 mEq/L to 10.9 ± 2.2 mEq/L (*p* < 0.001); and total weak acids from 16.1 ± 1.2 mmol/L to 14.0 ± 1.1 mmol/L (*p* < 0.001). The bicarbonate changed from 29.6 ± 2.7 mEq/L to 36.0 ± 4.1 mEq/L (*p* < 0.001); and lactate changed from 2.9 ± 1.5 mEq/L to 0.3 ± 0.03 mEq/L (*p* < 0.001) over time.

**Conclusions:**

The profound increase in the partial pressure of carbon dioxide that worsened during the total intravenous anaesthesia in both protocols initiated a substantial metabolic compensatory response to prevent severe acidaemia. This compensation resulted in a clinically acceptable mild acidaemic state, which worsened over time but not between the protocols, in healthy impala. However, these important compensatory mechanisms require normal physiological function and therefore when immobilising ill or anorexic wild ungulates their acid-base status should be carefully assessed.

## Background

Enzymes are important for metabolism and regulation of organ function and are labile to extreme acid-base changes, but operate efficiently within a narrow regulated pH range [1–3]. Research in patients demonstrating a pH shifts outside the narrow regulated range show decreased cardiac output and systemic vascular resistance and altered the oxygen binding to haemoglobin [1, 2, 4]. These cardiopulmonary observations may be applicable to the risks and success of anaesthesia and performance of wildlife ungulates on game farms.

Blood pH regulation is complex and involves various buffering systems and compensatory responses that keep the resultant pH within an optimal range for the species of animal [4–8]. Changes in pH are due to a change in the hydrogen ion (H^+^) concentration [6, 7].

The traditional Henderson-Hasselbalch approach and the Stewart physicochemical quantitative approach are used to interpret blood pH. The Henderson-Hasselbalch approach relates the blood pH to the constituents of the bicarbonate (HCO_3_^−^) buffering system (CO_2_ + H_2_O ↔ H_2_CO_3_ ↔ H^+^ + HCO_3_^−^) using the following equation [9, 10]:

$$ \mathrm{pH}=\mathrm{pKa}\ \mathrm{of}\ {\mathrm{H}}_2{\mathrm{CO}}_3+{\mathrm{log}}_{10}\ \left(\left[{{\mathrm{H}\mathrm{CO}}_3}^{-}\right]/\left[{\mathrm{H}}_2{\mathrm{CO}}_3\right]\right) $$

Which has been adapted for clinical application by the following equation [11, 12]:

$$ \mathrm{pH}={{\mathrm{pK}}_1}^{\hbox{'}}+{\mathrm{log}}_{10}\ \left(\left[{{\mathrm{HCO}}_3}^{-}\right]/S.{\mathrm{PCO}}_2\right) $$

where pK_1_´ is the equilibrium dissociation constant of carbonic acid = 6.105 at 37.0 °C (human); *S* is the solubility coefficient of carbon dioxide in plasma = 0.0307 [mmol/L]/mmHg.

The Stewart approach suggests that the HCO_3_^−^ and H^+^ represent the effect rather than the cause of acid-base derangements. Furthermore, the Stewart approach is based on the dissociation of water (H_2_O) to produce H^+^ or hydroxide ions (OH^−^) to maintain electrical neutrality within a solution (like blood) where there are independent variables (arterial partial pressure of carbon dioxide [PaCO_2_], strong ion difference [SID], anion gap [AG], total weak acids [Atot]) and dependent variables (H^+^, OH^−^, HCO_3_^−^, CO_3_^2−^, weak acids [HA] and ions [A^−^]) which influence the neutrality [13]. Any change in the independent variable will effect a change in the dependent variables to maintain electrical neutrality within the solution. Stewart’s theory has led to a revised version of the blood pH equation as follows [11, 12]:

$$ \mathrm{pH}={{\mathrm{pK}}_1}^{\hbox{'}}+\log \left\{\left(\left[{\mathrm{SID}}^{+}\right]-\mathrm{Ka}\left[\mathrm{Atot}\right]/\left(\mathrm{Ka}+{10}^{-\mathrm{pH}}\right)\right)/S.{\mathrm{PCO}}_2\right\} $$

where pK_1_´ is the equilibrium dissociation constant of carbonic acid; *S* is the solubility coefficient of carbon dioxide in plasma; Ka is the effective equilibrium dissociation constant of weak acids, the value is species dependent (Ka = 0.8 × 10^−7^ where pKa = 7.08; calves [11]). When using the Stewart approach the veterinarian must measure (PaCO_2_ using a blood gas analyser) or calculate the independent variables to help interpret the resultant blood pH. Strong ion differences, anion gaps and total weak acid concentrations may be calculated using frequently published equations in the veterinary literature (Table 1).

**Table 1:** Calculations used to calculate variables of interest to explain the acid-base balance in healthy impala (*Aepyceros melampus)* undergoing immobilisation and general anaesthesia using two different drug protocols

Variable	Equation used in study	Equation references	Unit	Ruminant values	Value references
SIDa	= ([Na^+^] + [K^+^] + [Ca^++^])-([Cl^−^] + [Lactate])	[4, 5, 37]	mEq/L	Calf: 39.3 ± 4.5	[18]
				Calf: 40.0 ± 2.0	[16]
SIDe	= 2.46 × 10^pH-8^ × PaCO_2_ + albumin (g/dL) × (0.123 × pH – 0.631) + phosphate (mEq/L) × (0.309 × pH – 0.469)	[4–6]	mEq/L	Calf: 34.8 ± 4.8	[18]
				Calf: 40.0 ± 2.0	[16]
SIG	= SIDa-SIDe	[5, 6]	mEq/L	Calf: 0.0 ± 3.0	[16]
AG	= ([Na^+^] + [K^+^])-([Cl^−^] + [HCO_3_^−^])	[4, 8, 14, 18]	mEq/L	Goat: 20.02 ± 0.5	[3]
				Goat: 12.62 ± 1.7	[17]
				Goat: 20.0 ± 3	[19]
				Goat: 17.1 ± 3.9	[14]
				Calf: 20.29 ± 4.5	[18]
Atot	= 2.25 × albumin (g/dL) + 1.4 × globulin (g/dL) + 0.59 × Phosphate (mg/dL)	[4, 5]	mmol/L	Calf: 18.2 ± 2.6	[18]
				Calf: 19.2 ± 6.1	[16]

*SIDa* apparent strong ion difference, *SIDe* effective strong ion difference, *SIG* strong ion gap, *AG* anion gap, *Atot* total weak acids in plasma, *Na* ^*+*^ sodium ion, *K* ^*+*^ potassium ion, *Ca* ^*++*^ calcium ion, *Cl* ^*−*^ chloride ion, *HCO*_*3*_^*−*^ bicarbonate ion, *g/dL* grams per decilitre, *mEq/L* milliequivilent per litre, *mg/dL* milligrams per decilitre, *mmol/L* millimoles per litre

There is a growing body of literature that provide reference ranges for the independent variables in domesticated production ungulates, in healthy [14] and diseased states [15–21]. However, there is a paucity of information regarding ranges of these variables in wildlife ungulates. Furthermore, the effect of various immobilisation and total intravenous anaesthesia protocols on blood pH balance have undoubtedly not been explored. Field ready drug protocols to maintain surgical anaesthesia in wild ungulates is becoming increasingly important, due to the increased demand of completing invasive surgical procedures such as bone fracture repair [22, 23]. The drug protocol should be made up of commonly available drugs and be easy to administer. Furthermore, the combination should maintain the animal’s organ physiology within clinically acceptable ranges to minimise compromising vital organ function [4, 5, 23].

The aims of this study were to measure and report the blood pH change over time in healthy adult female impala undergoing immobilisation and general anaesthesia using two different drug protocols. We hypothesise that blood pH would not be different between drug protocols and would not change over time. In addition, we aim to discuss the measured blood pH by describing the change in variables described by the Henderson-Hasselbalch and Stewart approaches of interpreting blood pH.

## Methods

This study was a part of a larger series of studies exploring the feasibility and cardiorespiratory effects of two different immobilisation and total intravenous anaesthetic protocols (drug protocols) administered for 120 min. All studies were approved by the animal ethics and research committees of the University of Pretoria prior to data collection (V099–13 & V012–16). The feasibility and cardiorespiratory outcomes of the two drug protocols are reported elsewhere and their findings are independent of those reported here [22, 23]. The present study reports on the acid-base status of the impala undergoing the two drug protocols.

Ten adult non-pregnant female impala aged between 12 and 36 months old were enrolled in this prospective cross-over study. The impala were captured from a nearby game farm and transported to the Faculty 6 weeks prior to the drug trials. They were housed in a purpose built 2.7 m high walled outdoor enclosure (boma) for the duration of the study. The boma was divided, by an internal wall with swing gates at either end, into a small area used for daily feeding and a larger home area. A 6 week pre-trial period was used to allow the impala to familiarise themselves with the boma and daily husbandry routine [24]. The impala received hay (*Erogrostis curvula*), lucerne (*Medecargo sative*) and water ad libitum; commercially available antelope pellets (Alzu antelope pellets; Alzu; South Africa; approximately 100 g/animal/day) were supplemented based on observed body condition.

All impala received two drug protocols (P-TMP & P-EME) on two occasions separated by 4 weeks:

- P-TMP – Immobilisation: thiafentanil (0.05 mg/kg; Thianil 1%; Wildlife Pharmaceuticals; South Africa) and medetomidine (0.055 mg/kg; Medetomidine 1%; Kyron Prescriptions; South Africa); Maintenance: propofol (12 mg/kg/h; Propoven 1%; Intramed, South Africa), ketamine (1.5 mg/kg/h; Ketamine Fresenius 10%; Intramed) and medetomidine (0.005 mg/kg/h; Domitor 0.1%; Zoetis; South Africa) ([22]).
- P-EME – Immobilisation: etorphine (0.05 mg/kg; Captivon 0.98%; Wildlife Pharmaceuticals) and medetomidine (0.055 mg/kg); Maintenance: etorphine (0.04 mg/kg/h), ketamine (1.5 mg/kg/h) and medetomidine (0.005 mg/kg/h) ([23]).

The impala were immobilised in the same order, on the same day of the week (two impala per day), at approximately the same time of the day, as randomised in the first week of data collection.

All impala were enclosed in the smaller feeding partition of the boma prior to darting. The impala were remotely injected using a filled dart (3 mL air pressurised dart; Dan-Inject; South Africa) containing the immobilisation combination, projected into the muscles of the pelvic girdle via a carbon dioxide powered rifle (set to 5 bar pressure, 12–15 m darting distance; Dan-Inject; Model JM). Once the dart was placed and fully discharged, a stopwatch was started to record the times to sampling. When the impala was immobilised into a recumbent position without attempts to stand the remaining impala were released into the larger home area of the boma and the immobilised impala was approached. An initial field clinical examination was completed and a cannula was aseptically placed into one of the cephalic veins prior to vehicle transport to the procedure room approximately 650 m away. Once in the procedure room, the impala was instrumented with a number of monitoring devices to measure cardiorespiratory and temperature parameters throughout the 120 min total intravenous anaesthesia ([22, 23]). Simultaneously, while placing the monitoring devices, an auricular artery was aseptically cannulated for serial arterial blood sampling and direct arterial blood pressure monitoring. The impala were left to breathe spontaneously throughout the study. If apnoea (no attempt to breathe over a 60 s period) was detected at any time during the procedures, then butorphanol (1:1 potent opioid dose) was administered intravenously [22, 23]. All impala tracheas were intubated (size 8.0 polyvinyl chloride cuffed endotracheal tube) and received oxygen insufflation (fixed rate of 2 L/min) via a nasogastric feeding tube (8 French Gauge; Avacare feeding tube; Sunray Medical; China) placed approximately to the level of the fourth intercostal space. Physiological saline (Sodium Chloride Fresenius 0.9%; Intramed; South Africa) was administered at a fixed maintenance rate of 5 mL/kg/h for the entire 120 min anaesthesia period.

Data collection of importance to the present study consisted of venous (lateral saphenous; needle and syringe technique; stored in serum tube) and arterial (aspirated from the auricular artery cannula using a pre-heparinised syringe and needle) blood sampling at two distinct time points. Time 1 was immediately prior to the start of the total intravenous anaesthesia infusion and oxygen supplementation, and Time 2 was 1 minute prior to cessation of total intravenous anaesthesia infusion, and before transporting the impala back to the boma for recovery. The times to sampling (from dart placement until sampling) for the two distinct times were recorded.

The venous sample was allowed to clot prior to centrifugation to separate the serum from the cellular components. The serum was carefully pipetted and stored in cryovials in a − 80 °C freezer until analysis. Serum phosphorus, albumin and globulin from the venous sample was analysed using a calibrated bench top serum analyser (Cobas, Integra 400 Plus; Roche Products (Pty) Ltd.; South Africa).

The arterial blood sample was collected and analysed immediately using a calibrated patient side blood gas analyser (EPOC Reader Blood Analysis Analyzer and EPOC BGEM smart cards; Epocal; USA). The blood gas analyser measured the following variables of interest: pH, PaCO_2_, sodium, potassium, calcium, chloride and lactate, haematocrit and haemoglobin concentration. The base excess (BE) and bicarbonate (HCO_3_^−^) was calculated based on the analyser’s internal algorithm setting for “other” species. All results were interpreted at a fixed body temperature of 37 °C (alpha-stat analysis). Rectal temperature (Physitemp Model BAT-12; Physitemp Instruments; USA) was continuously monitored and recorded at the time of blood sampling.

The impala were recaptured and transported back to their source on completion of the series of studies.

### Data analysis

Data were assessed for normality by plotting histograms, calculating descriptive statistics and performing the Anderson-Darling test for normality. Variables of interest (electrolytes, arterial carbon dioxide tension, base excess, bicarbonate, lactate, strong ion differences, anion gaps, total weak acids, proteins, haematocrit, haemoglobin concentration and temperature) were compared between protocols and time (both fixed effects) where impala were modelled as a random effect using a general linear mixed model analysis. Independent variables that cause the change in pH over time (partial pressure of carbon dioxide, apparent strong ion difference, anion gap and total weak acids) are presented graphically using box plots and whiskers [13]. Correlation between blood pH and variables of interest (bicarbonate ion, partial pressure of carbon dioxide, apparent strong ion difference, anion gap and total weak acids) were assessed using Persons correlation. The times to sampling for the first and second sampling points were compared between protocols using the two-sample t-test. Results reported as mean ± standard deviation (SD). Overall values were reported as mean ± standard deviation of the combined data from both protocols at the two time points. Data were analysed using commercially available statistical software (MiniTab 17.1.0; MiniTab Incorporated; USA) and results interpreted at the 5% level of significance. The main null hypothesis tested was that there would be no difference in blood pH between protocols and over time within a protocol.

## Results

The impala were weighed and the drug doses used for the immobilisation were recalculated on a per kilogram bases. In P-TMP, thiafentanil and medetomidine were dosed at 0.052 ± 0.007 and 0.057 ± 0.006 mg/kg, respectively. In P-EME, etorphine and medetomidine were dosed at 0.050 ± 0.012 and 0.054 ± 0.013 mg/kg, respectively. Both drug protocols immobilised the impala adequately. Within the first 15 min of recumbency butorphanol boluses were administered to eight impala in P-TMP and to three impala in P-EME that developed apnoea. Repeated butorphanol boluses were necessary in most impala receiving P-TMP. All impala were breathing regularly and spontaneously prior to Time 1 and no more butorphanol boluses were required.

One impala receiving P-EME sustained an inoperable comminuted fracture to a femur due to a darting injury and was humanly euthanised. Data collected from this impala were excluded from analysis.

The blood pH significantly decreased over time within both drug protocols (Table 2; *p* = 0.001), however, there was no significant difference between the two protocols at both the time points (*p* = 0.974; interaction: protocol x time). Overall, the pH changed from 7.37 ± 0.04 to 7.31 ± 0.05 at Time 1 to Time 2, respectively.

**Table 2:** Measured and calculated values obtained from healthy impala (*Aepyceros melampus)* undergoing immobilisation and general anaesthesia using two different drug protocols

Variable	Unit	Time 1				Time 2				*P* value
		P-TMP		P-EME		P-TMP		P-EME		
		Mean	±SD	Mean	±SD	Mean	±SD	Mean	±SD	
Times to sampling (from dart placement until sampling)										
Times to sampling	min	16.8	±7.0	19.2	±5.6	150.4	±5.7	151.8	±6.3	
T-test *P* value		*P* = 0.442				*P* = 0.613				
Basic clinical parameters at time of sampling										
Heart rate	Beats/min	122	±40	79	±37	57	±9	59	±11	<0.001
Resp rate	Breaths/min	9	±5	9	±2	10	±2	10	±2	0.316
MAP	mmHg	126	±14	117	±18	102	±12	90	±19	0.001
Temperature	°C	38.9	±0.4	39.3	±0.2	37.0	±0.2	37.0	±0.2	<0.001
Arterial blood acid base analysis										
pH	N/A	7.36	±0.04	7.38	±0.04	7.31	±0.02	7.30	±0.05	0.001
HCO_3_^−^	mEq/L	29.0	±2.7	30.1	±2.9	36.0	±5.9	35.9	±1.5	<0.001
	mmol/L	29.0	±2.7	30.1	±2.9	36.0	±5.9	35.9	±1.5	
BE	mEq/L	3.6	±2.4	4.9	±3.1	9.8	±6.1	9.5	±1.5	<0.001
	mmol/L	3.6	±2.4	4.9	±3.1	9.8	±6.1	9.5	±1.5	
Lactate	mEq/L	3.0	±1.6	2.9	±1.4	0.3	±0.0	0.3	±0.0	<0.001
	mmol/L	3.0	±1.6	2.9	±1.4	0.3	±0.0	0.3	±0.0	
Electrolytes										
Na^+^	mEq/L	145.8	±1.3	146.9	±2.3	146.4	±2.4	148.2	±2.4	0.170
	mmol/L	145.8	±1.3	146.9	±2.3	146.4	±2.4	148.2	±2.4	
K^+^	mEq/L	4.2	±0.2	4.1	±0.4	3.6	±0.1	3.6	±0.3	<0.001
	mmol/L	4.2	±0.2	4.1	±0.4	3.6	±0.1	3.6	±0.3	
Ca^++^	mEq/L	1.1	±0.1	1.1	±0.1	1.1	±0.1	1.1	±0.1	0.307
	mmol/L	0.55	±0.05	0.55	±0.05	0.55	±0.05	0.55	±0.05	
Cl^−^	mEq/L	107.4	±3.1	106.6	±2.8	103.9	±2.6	106.3	±2.4	0.046
	mmol/L	107.4	±3.1	106.6	±2.8	103.9	±2.6	106.3	±2.4	
P^−^	mEq/L	2.2	±0.4	2.2	±0.5	2.0	±0.5	2.2	±0.5	0.606
	mmol/L	2.2	±0.4	2.2	±0.5	2.0	±0.5	2.2	±0.5	
Proteins										
Albumin	g/dL	4.4	±0.3	4.2	±0.3	3.6	±0.2	3.7	±0.1	<0.001
Globulin	g/dL	1.8	±0.3	1.6	±0.3	1.5	±0.3	1.4	±0.2	0.003
Haematocrit	L/L	0.29	±0.03	0.28	±0.03	0.18	±0.02	0.20	±0.03	<0.001
Haemoglobin	g/dL	9.98	±0.86	9.42	±1.22	6.12	±0.72	6.72	±0.88	<0.001
Independent variables affecting pH										
PaCO_2_	mmHg	51.5	±8.6	51.2	±6.7	71.4	±15.6	73.8	±9.1	<0.001
SIDa	mEq/L	40.7	±1.9	42.7	±2.7	47.0	±4.3	46.2	±1.4	<0.001
	mmol/L	40.1	±1.9	42.1	±2.7	46.4	±4.3	45.7	±1.6	
SIDe	mEq/L	34.1	±3.0	35.1	±3.6	40.4	±6.0	40.8	±2.0	<0.001
	mmol/L	34.1	±3.0	35.1	±3.6	40.4	±6.0	40.8	±2.0	
SIG	mEq/L	6.6	±3.3	7.5	±1.8	6.5	±3.2	5.5	±2.2	0.243
	mmol/L	6.0	±3.2	7.0	±1.4	6.0	±3.2	5.0	±2.2	
AG	mEq/L	13.5	±4.0	14.3	±2.1	10.2	±2.8	9.5	±1.7	<0.001
	mmol/L	13.5	±4.0	14.3	±2.1	10.2	±2.8	9.5	±1.7	
Atot	mmol/L	16.4	±1.2	15.7	±1.2	13.8	±1.1	14.3	±1.0	<0.001

Time1: sampling prior to maintenance of general anaesthesia; Time 2: sampling 1 min prior to ending general anaesthesia; *P-TMP* protocol using thiafentanil-medetomidine immobilisation and propofol-ketamine-medetomidine infusion for general anaesthesia maintenance, *P-EME* protocol using etorphine-medetomidine immobilisation and etorphine-ketamine-medetomidine infusion for general anaesthesia, *P value* level of significance estimated over time, *min* minute, *Resp rate* respiratory rate, *MAP* direct mean arterial blood pressure, *HCO*_*3*_^*−*^ bicarbonate ion, *BE* base excess, *Na* ^*+*^ sodium ion, *K* ^*+*^ potassium ion, *Ca* ^*++*^ calcium ion, *Cl* ^*−*^ chloride ion, *P* ^*−*^ phosphorus ion, *PaCO*_*2*_ arterial partial pressure of carbon dioxide, *SIDa* apparent strong ion difference, *SIDe* effective strong ion difference, *SIG* strong ion gap, *AG* anion gap *Atot* total weak acids in plasma, *mmHg* millimetres mercury, *g/dL* grams per decilitre, *mEq/L* milliequivilent per litre, *mg/dL* milligrams per decilitre, *mmol/L* millimoles per litre

**Fig. 1:**
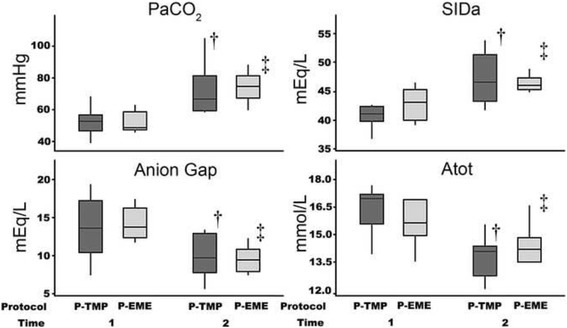
*Box plot* and whiskers of the independent variables in healthy impala (*Aepyceros melampus)* thought responsible for the change in hydrogen ion concentration (pH) in the plasma. Time 1 was sampling after immobilisation with either thiafentanil-medetomidine (P-TMP) or etorphine-medetomidine (P-EME). Time 2 was sampling at the end of either a propofol-ketamine-medetomidine (P-TMP) or an etorphine-ketamine-medetomidine (P-EME) total intravenous infusion. Where: PaCO_2_ is the arterial partial pressure of carbon dioxide; SIDa is the apparent strong ion difference; Atot is the total weak acid concentration in plasma; mmHg: millimetres mercury; mEq/L: milliequivence per litre and mmol/L: millimoles per litre; †: significant change in variable value over time for P-TMP protocol; ‡: significant

According to the Stewart approach, evaluation of the independent variables responsible for shifts of the hydrogen ion concentration, and thus blood pH, demonstrated statistically significant shifts over time that were of clinical interest (Fig. 1 and Table 2). The PaCO_2_ (*p* < 0.001) and SIDa (*p* < 0.001) increased, while the AG (*p* < 0.001) and Atot (*p* < 0.001) decreased over time. Yet, there was no significant difference between the two drug protocols for PaCO_2_ (*p* = 0.754), SIDa (*p* = 0.552), AG (*P* = 0.963) and Atot (*p* = 0.860). Overall, the independent variables changed, as follows: PaCO_2_ from 51.3 ± 7.5 mmHg to 72.6 ± 12.4 mmHg; SIDa from 44.6 ± 2.4 mEq/L to 46.9 ± 3.1 mEq/L; AG from 15.0 ± 3.1 mEq/L to 10.9 ± 2.2 mEq/L; and Atot from 16.1 ± 1.2 mmol/L to 14.0 ± 1.1 mmol/L at Time 1 to Time 2, respectively.

According to the Henderson-Hasselbalch approach, the PaCO_2_ (already described above) and serum bicarbonate are the variables of interest. Serum bicarbonate increased (*p* < 0.001) and serum lactate decreased (*p* < 0.001) over time, without significant differences between the two protocols at the time points (serum bicarbonate *p* = 0.676; serum lactate *p* = 0.782). Overall, the serum bicarbonate changed from 29.6 ± 2.7 mEq/L to 36.0 ± 4.1 mEq/L; and serum lactate changed from 2.9 ± 1.5 mEq/L to 0.3 ± 0.03 mEq/L at Time 1 to Time 2, respectively.

The blood pH demonstrated a strong negative correlation to the PaCO_2_ (*r* = −0.824; *p* < 0.001) and a moderate negative correlation to serum bicarbonate (*r* = −0.385; *P* = 0.020). The blood pH did not correlate to the SIDa (*r* = −0.316; *p* = 0.060) and Atot (*r* = 0.164; *p* = 0.341), respectively, but it did correlate moderately and positively to the AG (*r* = 0.413; *p* = 0.012).

The electrolytes did not change over time, with the exception of potassium (*p* < 0.001) and chloride (*p* = 0.046) which both decreased without a significant difference between the two protocols (potassium *p* = 0.192; chloride *p* = 0.398). Overall, potassium changed from 4.1 ± 0.3 mEq/L to 3.6 ± 0.3 mEq/L; and chloride change from 107.0 ± 2.9 mEq/L to 105.1 ± 2.7 mEq/L at Time 1 to Time 2, respectively.

The haematocrit (*p* < 0.001) and haemoglobin concentration decreased (*p* < 0.001) over time, without significant differences between the two protocols at the time points (haematocrit *p* = 0.856; haemoglobin concentration *p* = 0.944). Overall, the haematocrit changed from 0.29 ± 0.03 L/L to 0.19 ± 0.02 L/L; and haemoglobin concentration changed from 9.7 ± 1.1 g/dL to 6.4 ± 0.8 g/dL at Time 1 to Time 2, respectively.

The serum proteins, albumin (*p* = <0.001) and globulin (*p* = 0.003) significantly decreased over time with a significant differences between the two protocols for albumin (*p* = 0.036; interaction: protocol x time), yet, not for globulin (*p* = 0.092; interaction: protocol x time). Overall, the albumin changed from 4.3 ± 0.3 g/dL to 3.6 ± 0.2 g/dL; and globulin changed from 1.7 ± 0.3 g/dL to 1.5 ± 0.2 g/dL at Time 1 to Time 2, respectively.

## Discussion

The initial blood pH, after immobilisation, indicated a mild acidaemia (normal ruminant arterial blood pH reference range 7.37 to 7.48 [25]) due to the elevated PaCO_2_ causing a respiratory acidosis. Thereafter, the PaCO_2_ increased further and blood pH of the impala significantly decreased over time, regardless of the immobilisation and anaesthetic protocol used. The Henderson-Hasselbalch approach and the quantitative physicochemical Stewart approach were used to interpret the acid-base status of the impala. The profoundly elevated PaCO_2_ at the end of the anaesthesia would cause a respiratory acidosis, while the rising apparent strong ion difference (SIDa) and waning total weak acids (Atot) contributed to a simultaneous occurring metabolic alkalosis. Furthermore, the progressive elevation of the calculated serum bicarbonate (HCO_3_^−^) and base excess (BE) both indicate an emerging compensatory metabolic response. Therefore the resultant pH values at the end of the anaesthesia are because of a pronounced metabolic compensatory response to the severe respiratory acidosis that resulted in an overall mild acidaemia. Because there are no published reference ranges for acid-base variables in resting impala we used ranges from closely related species (healthy awake goats and calves; Table 1) to interpret our findings.

Unfortunately, more advanced techniques used to calculate compensation, like expected compensatory changes in PaCO_2_ or bicarbonate ion concentrations, would be difficult to use for interpretation due to the paucity in referenced normal ranges for small wild ungulates. Although the impala were habituated to the boma, they were not tame enough for us to obtain awake control or reference samples. Gaining such samples from an awake wild animal can only be achieved by using remote sampling devices [26]. Such devices are not readily available and need to be custom made per species [27].

We expected respiratory acidosis to be pronounced, especially at the end of the anaesthesia, as PaCO_2_ was greatly elevated compared to the normal awake range of 35–45 mmHg in mammals [5]. The PaCO_2_ at the end of the anaesthesia was substantially higher compared to just after induction into immobilisation in our impala, and compared to values measured in other immobilised impala (PaCO_2_ of 39.1 ± 3.4 to 41.3 ± 5.0 mmHg) [28]. One of the stimuli to take a breath in a healthy awake animal is brought about by the rising PaCO_2_ level reaching a threshold. The drugs used in this study, especially the potent opioids, either alone or in combination with the other anaesthetic and sedative drugs, are known to cause respiratory-neuronal depression [29, 30] which shifts the carbon dioxide respiratory response curve to the right [31]. Whereby a higher threshold level of PaCO_2_ is required to stimulate the respiratory centre to initiate a breath. Therefore these drugs ultimately result in hypoventilation (decreased alveolar minute ventilation) which causes the increase in PaCO_2_. Ventilation is challenging to assess when only subjectively monitoring the respiratory system by counting the respiratory rate and estimating the tidal volume. Often an animal will appear to be ventilating normally, as in the case of these impala that had a normal respiratory rate and tidal volume at Time 1, after dosing with butorphanol (data reported elsewhere) [22, 23], but on closer examination this may not be the case. Thus, more invasive monitoring tools, such as arterial blood gas analysis or capnography, may be required to detect shifts in blood pH that are due to alterations in ventilation [32]. Furthermore, other co-aetiologies should always be considered when there is an obvious respiratory acidosis without overt evidence of simple hypoventilation, such as severe right-to-left pulmonary shunting, large dead-space ventilation or ventilation-perfusion mismatch [5, 23, 30]. Haemoglobin is an important intracellular buffer that will bind reversibly to either carbon dioxide or to the hydrogen ion formed by the bicarbonate buffer system, to transport them from the metabolising tissues to the lungs [4, 5, 8, 33, 34]. With oxygen supplementation an increase in the PaO_2_ will increase the force for oxygen to bind to haemoglobin as opposed to carbon dioxide or hydrogen ions (Haldane Effect; high PaO_2_ levels decrease the buffering effects of haemoglobin, therefore hydrogen ions are unbound from haemoglobin to preferentially transport oxygen). Therefore, during anaesthesia, the PaCO_2_ in the impala most likely increased due to the oxygen supplementation [4, 5, 33, 34]. Furthermore, the haemoglobin concentration (and haematocrit) dropped during general anaesthesia [35], a known phenomenon in patients under general anaesthesia, especially if alpha2-adrenoceptor agonists like medetomidine are used [36], therefore decreasing an important plasma buffering system which could have also contributed to the increase in PaCO_2_ and hydrogen ion concentration. The emergency treatment of apnoea with butorphanol did result in a regular spontaneous breathing pattern in the impala immobilised with both protocols [22, 23]. A limitation to this study is that we did not take arterial blood samples immediately after recumbency, while apnoeic episodes occurred, especially in P-TMP which required more frequent butorphanol boluses compared to P-EME. Therefore, the effects of butorphanol on the blood pH could not be determined. However, in another etorphine immobilized ungulate, the goat, butorphanol corrected hypoxaemia but not hypercapnia and therefore it may be that it had little influence on blood pH in the impala at Time 1 [37].

Irrespective of the cause of the respiratory acidosis, metabolic compensation, indicated by the significant rise in the bicarbonate ion concentration, occurred within a 120 min. This indicator of compensation is according to the traditional Henderson-Hasselbalch approach used to evaluate blood pH, whereby the body attempts to correct the increased hydrogen ion concentration by elevating the bicarbonate ion concentration to normalise the bicarbonate ion to carbonic acid ratio ([HCO_3_^−^]:[H_2_CO_3_] ratio) back to 20:1 [4]. The PaCO_2_ corresponds to H_2_CO_3_ and is merely substituted to simplify the calculation of the compensatory response [12, 17, 28]. Therefore, any rise in PaCO_2_ should be met by a rise in the HCO_3_^−^ ions in uncomplicated respiratory acidosis, as demonstrated in these impala.

A simple change in the HCO_3_^−^ does not completely explain the acid-base compensation that occurred in the impala. The apparent strong ion difference (SIDa) was higher and the total weak acids (Atot) were lower than that of published ranges for healthy control goats and calves. Both changes indicate an additional non-respiratory alkalinising effect [5–7, 11]. The measured electrolytes (sodium, potassium, calcium, chloride and phosphorus) were within accepted published ranges for impala [38]. Furthermore, all but the potassium and chloride concentration did not significantly change over time. Yet the decrease in the potassium level was not large enough to solely explain the increased apparent strong ion difference (SIDa) value. Therefore, the decrease in lactate ion concentration that occurred most likely contributed the most to the increased apparent strong ion difference (SIDa) at the 120 min measurement. The drop in the chloride concentration could have been due to the increase in plasma bicarbonate, whereby the plasma attempts to maintain electrical neutrality by excreting chloride [5–7, 12, 39]. At the rate of administration used it is unlikely that the infused physiological saline increased sodium or chloride concentrations in the plasma of the impala, as reported in healthy dogs [40]. The decreased total weak acids (Atot) over time was attributed to the decrease in albumin and globulin concentrations. A decrease in plasma protein levels has been described in animals undergoing general anaesthesia [35], especially when alpha2-adrenoceptor agonists such as medetomidine are administered [36]. The total weak acids (Atot) levels reported in this study were also lower compared to goats and calves. This difference could be attributed to different measurements of phosphorus, or different calculations used to determine the total weak acids (Atot). Furthermore, the rising bicarbonate ion concentration could cause the negatively charged proteins to move out of the plasma in order to maintain electrical neutrality, a plausible theory requiring further confirmation.

The decrease in the anion gap (AG) over time was attributed to the pronounced increase in the HCO_3_^−^ concentration. Overall the anion gap (AG) was substantially lower than those reported for goats and calves [14, 17–20]. However, the HCO_3_^−^ concentrations and BE at the end of the anaesthesia were higher than a generally accepted upper limit of 30 mmol/L and 6.0 to 8.0 mmol/L, respectively for herbivores [4, 5, 8] and the published ranges for healthy goats [14, 17, 19] and calves [18, 20]. Both of these variables demonstrate that a metabolic compensatory response was initiated to correct the respiratory acidosis in the impala.

The worsening acidaemia, and precipitous drop in serum protein concentrations, especially albumin, may alter ionisation and protein binding of drugs, which could have profound effects on drug pharmacokinetics and dynamics [41]. These possible alterations warrant further investigation to gain better clarity of their clinical implications. In other words, did the impala in this study experience more pronounced respiratory depression due to alterations in the pharmacokinetics and dynamics of the drugs used to maintain general anaesthesia by causing a relative overdose?

Furthermore, the clinical implications of acidosis, regardless of cause, are serious and warrant careful consideration. The effects of acidosis on the cardiovascular system include negative inotropy, tachycardia and vasodilation which translates into a decreased blood pressure due to the reduction in cardiac output (decreased stroke volume) and systemic vascular resistance [1, 2]. Oxygen binding to haemoglobin is altered, causing a right shift in the oxygen-haemoglobin dissociation curve (Bohr Effect; acidaemic plasma pH, usually brought about by increasing PaCO_2_ levels at metabolically active tissue decrease haemoglobins affinity for oxygen, therefore increase its offloading to the tissue) [4, 5]. The right shift translates into a decrease affinity for haemoglobin to bind to oxygen, therefore less is transported to the tissue potentially resulting in tissue hypoxia. Among other causes, the decrease in pH increases the stimulation to breathe which results in increased workload of the respiratory system and therefore global oxygen demand. Furthermore, the decrease in cardiovascular performance decreases oxygen delivery [2]. Animals that cannot initiate compensatory responses to acidosis due to illness or the effects of anaesthetic drugs or both may suffer further physiological derangements which could lead to increased morbidity or mortality.

The shift in blood pH was evident in healthy impala undergoing immobilisation and general anaesthesia using two different drug protocols. The profound increase in PaCO_2_, despite a seemingly normal respiratory rate for a medium sized ruminant suggests that monitoring for a respiratory acidosis is not reliable when using just the respiratory rate as an indicator of respiratory suppression. The gases found within the alveoli while breathing room air include nitrogen, oxygen and carbon dioxide (originating from the metabolically active tissue and transported to alveoli via the blood). If the amount of carbon dioxide increases to levels above 50 mmHg, as noted in these impala, then there is an increased competition for gases within the alveoli [4, 5]. This competition decreases the amount of oxygen that is available for absorption and can lead to hypoxaemia [4, 5]. Therefore, if these protocols are to be used in the field, oxygen supplementation should be considered mandatory [22, 23]. Despite the substantial increase in PaCO_2_ over time the acidaemic shift in the blood pH was negligible due to the profound compensatory metabolic responses that we detected. These important responses require normal health and physiological function and therefore caution should be taken, and acid-base status carefully assessed, when immobilising ill or anorexic wild ungulates.

## Conclusion

The profound increase in the partial pressure of carbon dioxide that worsened during the total intravenous anaesthesia in both protocols initiated a substantial metabolic compensatory response to prevent severe acidaemia. This compensation resulted in a clinically acceptable mild acidaemic state, which worsened over time but not between the protocols, in healthy impala. However, these important compensatory mechanisms require normal physiological function and therefore when immobilising ill or anorexic wild ungulates their acid-base status should be carefully assessed. In addition, whenever impala are immobilised with thiafentanil or etorphine based drug combination respiration should be closely monitored and butorphanol and oxygen supplementation should be considered in apnoea and hypoxia occurs.

## Abbreviations

AG: Anion gap
Atot: Total weak acids
H^+^: Hydrogen ion
HCO_3_: Bicarbonate ion
PaCO_2_: Arterial partial pressure of carbon dioxide
PaO_2_: Arterial partial pressure of oxygen
SID: Strong ion difference
